# The Influence of Bariatric Surgery on Reproductive Hormones and Ovarian Morphology and Clinical Findings in Women: A Prospective Study

**DOI:** 10.1007/s11695-025-08012-2

**Published:** 2025-06-24

**Authors:** Yagmur Soykan, Hüseyin Bayhan, Serkan Akogul, Abdulkadir Bedirli

**Affiliations:** 1 Gazi University, Ankara, Turkey; 2 Pamukkale University, Denizli, Turkey

**Keywords:** Bariatric surgery, Ovarian hormones, Adrenal androgens, Hirsutism, Dysmenorrhea, Polycystic ovary

## Abstract

**Background:**

Obesity in women of reproductive age often causes hormonal imbalances and fertility issues. Bariatric surgery effectively promotes weight loss and metabolic improvement, but its impact on reproductive hormones warrants further investigation. This study examines bariatric surgery’s effects on hormonal and clinical changes in women preoperatively and at 3 and 6 months postoperatively.

**Methods:**

This prospective study included 67 women undergoing bariatric surgery. Anthropometric measurements, hormonal profiles, ovarian morphology(via ultrasonography), and the presence of hirsutism and dysmenorrhoea were evaluated preoperatively and at 3- and 6-month follow-ups.

**Results:**

Bariatric surgery resulted in significant weight loss and hormonal changes. Body weight (*p* < 0.001) and BMI (*p* < 0.001) decreased substantially, with the most rapid reduction occurring in the first 3 months, followed by a slower decline. Androstenedione levels decreased significantly (*p* < 0.001). Total testosterone levels showed a significant reduction at 6 months (*p* < 0.001), while SHBG (*p* < 0.001; *p* = 0.014), DHEA-S, and AMH (*p* < 0.001; *p* < 0.001; *p* = 0.014) levels increased. No significant changes were observed in FSH and LH levels (*p* > 0.05). The severity of dysmenorrhea decreased significantly at 6 months (*p* < 0.001). Additionally, the prevalence of hirsutism (*p* < 0.001) and polycystic ovary (PCO) morphology (*p* < 0.001) decreased significantly at 6 months.

**Conclusions:**

Bariatric surgery significantly decreases testosterone and androstenedione, while increasing AMH, DHEA-S, and SHBG. Improvements in PCO morphology, dysmenorrhea, and hirsutism highlight the effects of bariatric surgery on hormonal balance and reproductive health.

## Introduction

Obesity is a growing global health concern, with its increasing prevalence linked to hormonal imbalances, metabolic disorders, and adverse effects on reproductive function [1]. In women of reproductive age, obesity significantly disrupts hormonal balance, particularly in the ovarian and adrenal glands. Ovarian hormones—such as estrogen, progesterone, and testosterone—are essential for reproductive health and overall well-being. Additionally, adrenal hormones—including cortisol, dehydroepiandrosterone sulfate (DHEA-S), and androgens—play key roles in metabolism, stress response, and sex hormone production [2]. By disturbing the balance of these hormones, obesity markedly increases the risk of complications such as polycystic ovary syndrome (PCOS), infertility, pregnancy-related issues, and cardiovascular diseases in women of reproductive age.

Bariatric surgery is one of the most effective treatments for morbid obesity and is considered an advanced therapeutic option. Various bariatric surgery techniques—including gastric bypass, sleeve gastrectomy, and adjustable gastric banding—promote weight loss through different mechanisms, leading to significant metabolic improvements. These surgical procedures facilitate weight loss and improve obesity-related comorbidities, such as type 2 diabetes (T2D), through complex mechanisms beyond simple caloric restriction and malabsorption. These include favorable alterations in gut hormone secretion (e.g., increased GLP-1 and PYY), changes in bile acid profiles, modulation of the gut microbiome, and potentially increased energy expenditure [3]. These physiological changes contribute to enhanced insulin sensitivity and glucose homeostasis, and may exert beneficial effects on reproductive health. Indeed, the positive impact of bariatric surgery on reducing infertility and improving pregnancy outcomes in obese women has been previously demonstrated through systematic reviews and meta-analyses [4, 5]. These studies highlight that weight loss following bariatric surgery contributes to the improvement of ovulatory dysfunction and irregular menstruation, thereby increasing spontaneous conception rates and reducing miscarriage rates [4, 5]. Similarly, Babarinsa et al. have reported that bariatric surgery has positive effects on fertility and sexuality[6].

Postoperative weight loss has been associated with notable hormonal changes. In particular, improvements in PCOS symptoms—such as reduced insulin resistance, lower androgen levels, regulated ovulation, and alleviated menstrual irregularities—have been observed. Furthermore, bariatric surgery has been linked to improved pregnancy outcomes [7].

However, the hormonal effects of bariatric surgery are complex, and ongoing research aims to further elucidate these mechanisms. Key areas requiring clarification include long-term hormonal changes following bariatric surgery and variations in outcomes based on individual patient characteristics.

This study aims to comprehensively evaluate the effects of bariatric surgery on clinical, biochemical, and radiological outcomes in women of reproductive age. Specifically, it will analyze weight loss, ovarian and adrenal hormone levels, ovarian morphology, and clinical findings following bariatric surgery.

## Materials and Methods

The study included female patients aged 18 to 49 years with a body mass index (BMI) of 35 and higher who were scheduled to robotic bariatric surgery by the General Surgery Clinic. Participants were selected from those referred to the Gynaecology and Obstetrics Clinic for gynecological complaints during the preoperative evaluation process. Demographic data, medical history, and gynecological history were recorded for all patients meeting the inclusion criteria.

Patients using hormonal contraceptives (due to their influence of sex steroid hormone levels) and those with a history of ovarian surgery (due to its impact on Anti-Müllerian Hormone [AMH] levels) were excluded from the study. Blood samples were collected at three time points: preoperatively, and at 3 and 6 months postoperatively, for hormonal analysis. The levels of Follicle-Stimulating Hormone (FSH), Luteinizing Hormone (LH), Sex Hormone-Binding Globulin (SHBG), total testosterone, androstenedione, DHEA-S, and AMH were measured.

The severity of hirsutism was assessed using the Ferriman-Gallwey score, while the severity of dysmenorrhea was evaluated based on the patient’s self-reported pain score as determined by a numeric rating scale (NRS)[8]. Polycystic ovary (PCO) was diagnosed via ultrasound. It is characterized by the presence of hyperechoic central stroma, peripheral follicle distribution (string of pearls sign), and multiple follicles of uniform size (diameter: 2–9 mm).

All patients were closely monitored throughout the study period. Blood samples were collected preoperatively and at 3 and 6 months postoperatively for hormonal assessments, and gynecological examinations were conducted at each time point. Participants whose blood samples could not be obtained at the scheduled time points or whose hormone levels could not be evaluated were excluded from the study.

### Statistical Analysis

Statistical analyses were performed using IBM SPSS Statistics 29 software. Categorical variables were presented as with frequency and percentage (%), while numerical variables were reported as mean ± standard deviation and median (min–max) values.

Kolmogorov–Smirnov normality test was used to assess the distribution characteristics of continuous variables. Nonparametric analysis methods were applied for data that did not follow a normal distribution. To analyze changes in dependent numerical variables over time, the non-parametric Friedman test was employed for repeated measures analysis. If a significant difference was detected post hoc Wilcoxon tests and Bonferroni correction were conducted to determine which time points differed.

For binary categorical dependent variables measured over time, Cochran’s *Q* test was used to assess differences. If significant results were obtained, the post hoc McNemar test and Bonferroni correction were applied. A *p*-value of < 0.05 was considered statistically significant in all analyses.

## Results

A total of 67 female patients participated in this study. Variables were measured preoperatively and at 3 and 6 months postoperatively in women with a BMI of 35 or higher The distribution of these variables is summarized in Table 1.

**Table 1:** Distribution of findings regarding the study

Variable	*n*	%
Type of surgery		
Robotic sleeve gastrectomy	16	23.9
Robotic Roux-en-Y gastric bypass	51	76.7
Disease		
None	45	67.2
Present	22	32.8
Type of disease		
None	45	67.2
Diabetes mellitus (DM)	7	10.4
Hypothyroidism	6	9
Asthma	4	6
Hypertension (HT)	1	1.5
HT + DM	3	4.5
HT + DM + hypothyroidism	1	1.5
Education status		
Primary school	9	13.4
Secondary school	5	7.5
High school	29	43.3
University	24	35.8
Parameter	Mean ± SS	Median [min–max]
Age (year)	32.72 ± 8.49	31 [18–49]
Heights (meters)	1.63 ± 0.06	1.63 [1.50–1.78]
Weights (kg)	112.34 ± 19.32	109 [80–165]

According to Table 1, 76.7% of patients underwent robotic Roux-en-Y gastric bypass surgery, while 23.9% underwent robotic sleeve gastrectomy. Comorbidities were present in 32.8% of participants, with the most common being diabetes (10.4%), hypothyroidism (9.0%), asthma (6.0%), and hypertension (1.5%). The remaining 67.2% had no comorbidities. Regarding educational background, 43.3% of patients were high school graduates, 35.8% held a university degree, 13.4% had completed primary school, and 7.5% had completed secondary school. The mean age of participants was 32.72 ± 8.5 years, with an average height of 1.63 ± 0.06 m and a mean weight of 112.34 ± 19.32 kg.

In this study, preoperative, 3rd-month, and 6th-month measurements of weight, BMI, FSH, LH, total testosterone, AMH, DHEA-S, SHBG, and androstenedione and dysmenorrhea were evaluated using the Kolmogorov–Smirnov test, which indicated that these variables were not normally distributed (*p* < 0.05). Accordingly, the nonparametric Friedman test was used to analyze the changes over time. For variables showing significant differences in the Friedman test, post hoc Wilcoxon tests with Bonferroni corrections were applied to determine differences between time points. The results of these analyses are presented in Table 2.

**Table 2:** Friedman test results of repeated measures of variables

	Follow-up	Mean ± SS	Median [min–max]	Friedman test	Post hoc Bonferroni
Weight (kg)	*Preop* *3rd month* *6th month*	112.34 ± 19.32 87.12 ± 13.70 71.66 ± 11.40	109 [80–165] 83 [68–126] 68 [54–98]	*χ*^2^ = 134 ***p* < 0.001**	Preop > 3rd month Preop > 6th month 3rd month > 6th month
BMI (kg/m^2^)	*Preop* *3rd month* *6th month*	42.25 ± 7.74 32.78 ± 5.60 26.97 ± 4.67	41 [30.6–71.1] 31.5 [23.4–55.6] 26.3 [19.4–43.6]	*χ*^2^ = 134 ***p* < 0.001**	Preop > 3rd month Preop > 6th month 3rd month > 6th month
Androstenedione	*Preop* *3rd month* *6th month*	2.41 ± 1.29 2.26 ± 1.23 2.06 ± 0.97	2.22 [0.30–7.25] 2 [0.26–6.60] 1.87 [0.43–5.70]	*χ*^2^ = 31.731 ***p* < 0.001**	Preop > 3rd month Preop > 6th month 3rd month > 6th month
Total testosterone	*Preop* *3rd month* *6th month*	0.58 ± 0.30 0.53 ± 0.30 0.45 ± 0.26	0.54 [0.10–1.29] 0.46 [0.10–1.54] 0.38 [0.10–1.16]	*χ*^2^ = 45.608 ***p* < 0.001**	Preop = 3rd month Preop > 6th month 3rd month > 6th month
SHBG	*Preop* *3rd month* *6th month*	35.54 ± 31.47 38.04 ± 32.33 46.32 ± 33.80	26.5 [9.3–186] 32 [8.2–198] 43 [8.60–189]	*χ*^2^ = 56.448 ***p* < 0.001**	Preop = 3rd month Preop < 6th month 3rd month < 6th month
DHEA-S	*Preop* *3rd month* *6th month*	229.72 ± 133.45 237.46 ± 123.90 249.43 ± 124.07	206 [14–511] 230 [22–467] 244 [44–486]	*χ*^2^ = 25.642 ***p* < 0.001**	Preop = 3rd month Preop < 6th month 3rd month < 6th month
AMH	*Preop* *3rd month* *6th month*	3.63 ± 3.91 3.82 ± 4.11 4.03 ± 4.27	2.89 [0.1–21.5] 2.84 [0.1–22] 3.00 [0.1–22.5]	*χ*^2^ = 8.475 ***p* = 0.014**	Preop = 3rd month Preop < 6th month 3rd month = 6th month
FSH	*Preop* *3rd month* *6th month*	8.64 ± 4.16 8.59 ± 4.28 8.40 ± 4.40	7.8 [1.71–22.32] 8 [2–21.8] 7.86 [1.98–24]	*χ*^2^ = 2.239 *p* = 0.326	-
LH	*Preop* *3rd month* *6th month*	7.57 ± 4.07 7.70 ± 4.22 7.77 ± 5.03	6.82 [0.71–20.82] 7 [1.6–22] 6.7 [1.24–27.49]	*χ*^2^ = 2.179 *p* = 0.336	-
Dysmenorrhoea	*Preop* *3rd month* *6th month*	3.64 ± 3.03 2.67 ± 2.57 1.66 ± 2	4 [0–10] 2 [0–10] 1 [0–8]	*χ*^2^ = 76 ***p* < 0.001**	Preop > 3rd month Preop > 6th month 3rd month > 6th month

Bold values indicate statistically significant results (*p *<0.05)

Significant reductions in both weight and BMI were observed following surgery (*p* < 0.001), with the most rapid decline occurring between the preoperative period and 3 months post-surgery. This was followed by a slower yet still significant decrease from 3 to 6 months. These changes were statistically significant at all time points compared to baseline, as well as between the three- and six-month measurements.

A statistically significant difference in androstenedione levels was observed among the preoperative, 3-month, and 6-month measurements (*p* < 0.001). Compared to preoperative levels, mean androstenedione levels decreased significantly at 3 and 6 months. The reductions between the preoperative period and postoperative time points were statistically significant, as was the decrease between the 3rd and 6th months.

A statistically significant difference in total testosterone levels was observed among the preoperative, 3-month, and 6-month measurements (*p* < 0.001). While no significant difference was found between the preoperative and 3-month levels, total testosterone levels significantly decreased by the 6th month compared to both the preoperative period and the 3rd month.

A statistically significant increase in SHBG levels was observed between the preoperative period and the 3rd and 6th months (*p* < 0.001; *p* = 0.014). While no significant difference was found between the preoperative and 3rd-month levels, SHBG levels at the 6th month were significantly higher than those in both the preoperative period and the 3rd month.

A statistically significant difference in DHEA-S and AMH levels was observed between the preoperative period and the 3rd and 6th months (*p* < 0.001; *p* < 0.001; *p* = 0.014). Mean DHEA-S and AMH levels increased between the 3rd and 6th months compared to preoperative levels. While no statistically significant difference was found between the preoperative period and the 3rd month, levels at the 6th month were significantly higher than both the preoperative and 3rd-month values.

No statistically significant differences were observed in FSH and LH levels among the preoperative, 3rd-month, and 6th-month measurements (*p* > 0.05). Mean FSH and LH levels at 3 and 6 months post-bariatric surgery remained similar to preoperative values.

In the analysis of dysmenorrhea severity, statistically significant differences were found between preoperative, three-month, and six-month data (*p* < 0.001). Compared to preoperative levels, there was a significant decrease in mean dysmenorrhea severity at 3 and 6 months. The differences between the preoperative period and the 3rd and 6th months were statistically significant, indicating that dysmenorrhea severity decreased notably between the 3rd and 6th months.

In this study, preoperative, 3-month, and 6-month data were compared to assess the effects of bariatric surgery on hirsutism (absent/present) and polycystic ovary (PCO) appearance**.** Cochran’s *Q* test was used for statistical analysis, and in cases of significant results, post hoc McNemar test with Bonferroni correction was applied. The findings from these analyses are presented in Table 3**.**

**Table 3:** Cochran’s *Q* test results of variables

	Follow-up	Negative *N* (%)	Positive *N* (%)	Cochran’s *Q* test	Post hoc Bonferroni
Hirsutism	*Preop* *3rd month* *6th month*	51 (%77) 51 (%77) 58 (%87)	16 (%23) 16 (%23) 9 (%13)	*Q* = 14 ***p* < 0.001**	Preop = 3rd month Preop ≠ 6th month 3rd month ≠ 6th month
PCO morphology	*Preop* *3rd month* *6th month*	38 (%57) 43 (%65) 49 (%74)	29 (%43) 24 (%35) 18 (%26)	*Q* = 16.55 ***p* < 0.001**	Preop = 3rd month Preop ≠ 6th month 3rd month = 6th month

Bold values indicate statistically significant results (*p* <0.05)

A statistically significant difference was observed in the prevalence of hirsutism between preoperative data and the measurements taken at 3 months and 6 months (*p* < 0.001). Post hoc analyses indicated that the differences between the preoperative period and the 6-month data, as well as between the 3-month and 6-month data, were significant (*p* < 0.05); however, no significant difference was found between the preoperative period and the 3-month data (*p* > 0.05). Overall, the change in hirsutism following bariatric surgery did not show significant improvement until the 3-month mark, but a noteworthy decrease was observed by the 6-month point. These findings suggest that the effect of bariatric surgery on hirsutism increases over time, providing a statistically significant reduction, particularly at 6 months.

A statistically significant difference was found in the frequency of PCO appearance between the preoperative period, as well as the 3-month and 6-month data (*p* < 0.001). Post hoc analyses indicated a significant difference between the preoperative period and the 6th month (*p* < 0.05); however, no significant difference was noted between the preoperative period and the 3rd month or between the 3rd month and the 6th month (*p* > 0.05). Overall, a decrease in the appearance of PCO was noted in the first 3 months after bariatric surgery, but no significant change was observed, while a significant decrease occurred by the 6th month compared to the preoperative period.

## Discussion

As research on hormonal changes following bariatric surgery continues to expand, our study makes a unique contribution to the literature by incorporating ultrasonographic evaluation of ovarian morphology and examining gynecological clinical findings, alongside a comprehensive hormonal analysis.

Excess body fat in obese patients can disrupt the hypothalamic-pituitary–gonadal (HPG) axis through both central and peripheral mechanisms [9]. Dysregulation of this axis can lead to anovulation, infertility, and menstrual irregularities [7]. Weight loss is believed to have beneficial effects by regulating the menstrual cycle, enhancing ovulatory function, and improving fertility in obese women [4]. As a result, bariatric surgery is recommended for patients with a body mass index (BMI) ≥ 40 kg/m^2^ or a BMI of 35–39.9 kg/m^2^ with comorbidities, particularly when conventional interventions such as diet, exercise, and pharmacological treatments have been unsuccessful [10].

Although the literature presents varying findings on the effects of bariatric surgery on the hormonal axis, our study revealed no significant changes in FSH and LH levels. Specifically, FSH and LH levels remained stable between preoperative measurements and those taken at 3 and 6 months postoperatively. Previous studies have reported inconsistent results regarding FSH and LH levels after bariatric surgery. In a study by Santaro et al., obese women were found to have irregular and prolonged menstrual cycles, along with decreased urinary levels of luteinizing hormone (LH), follicle-stimulating hormone (FSH), estrogen metabolites, and progesterone [11]. Conversely, Emami et al. reported that after bariatric surgery, LH, FSH, and SHBG levelssignificantly increased, while estradiol (E2) levels decreased in women [12]. Similarly, in a study by Paul et al., estradiol, progesterone, LH, and FSH levels remained unchanged compared to preoperative values [13]. However, Anbara et al.found that FSH and LH levels increased at 3 and 6 months postoperatively, while estrogen and progesterone levels remained unchanged at 3 months after surgery [14]. These inconsistencies may be due to the fact that FSH and LH are less affected by weight loss following bariatric surgery. Increased adipose tissue in obesity enhances aromatase enzyme activity, leading to higher estrogen production. It is suggested that the variability in estradiol levels across studies may stem from individual patient differences.

Sex Hormone Binding Globulin (SHBG) is a circulating protein that transports sex hormones in the blood, primarily testosterone and estradiol, the two most critical sex hormones. However, SHBG can also bind to other steroid hormones, such as dihydrotestosterone (DHT) and androstenedione, albeit with lower affinity. Excess weight, particularly visceral fat (fat surrounding the abdominal organs), is a major contributor to insulin resistance. Following bariatric surgery, weight loss leads to a decrease in insulin resistance. Consequently, SHBG (sex hormone-binding globulin) levels increase, binding more testosterone and thereby reducing the amount of free, biologically active testosterone. Bariatric surgery has been shown to decrease androstenedione and total testosterone levels in women [12]. In our study, we observed a statistically significant increase in SHBG levels at 6 months postoperatively, following weight loss. Consistent with previous findings, our study observed a significant reduction in total testosterone and androstenedione levels as weight loss progressed. These results further support the role of bariatric surgery in lowering androgen levels. Similarly, in a study involving 100 women after gastric bypass, a significant drop in testosterone levels was observed alongside a notable increase in SHBG [13]. Additionally, in the study by Lv et al., bariatric surgery led to decreased levels of total testosterone, DHEA, and estradiol, while SHBG levels increased, a change that was associated with a reduction in the incidence of abnormal menstruation [15]. The observed increase in SHBG and decrease in androgen levels following bariatric surgery underlines the importance of understanding the hormonal effects of this procedure and emphasizes the need for additional replacement therapy tailored to individual needs.

Our study evaluated ovarian morphology via ultrasonography during the preoperative period and at 3 and 6 months postoperatively. During the preoperative assessment, 43% of the women exhibited ultrasonographic signs of PCO. While no significant change in ovarian morphology was observed at 3 months postoperatively, the proportion of women with PCO appearance decreased to 27% at the 6th-month follow-up. The absence of a statistically significant change at 3 months suggests that the effects of hormonal changes and weight loss on ovarian morphology may take longer to manifest, indicating no substantial structural improvement in the short-term. Similarly, a study by Singh et al. reported PCO in 77% of women (14 out of 18) before bariatric surgery, with complete recovery observed in 55% of these women during the one-year postoperative follow-up [16]. Various studies have demonstrated that bariatric surgery reduces ovarian volume in women with PCOS. In a study conducted on obese and infertile women with PCOS, bariatric surgery was shown to restore physiological balance by significantly reducing ovarian volume, a finding supported by ultrasonographic evaluations [17]. A gradual reduction in ovarian morphology and volume was observed, with the mean ovarian volume decreasing from 14.7 ± 1.79 ml to 9.78 ± 1.19 ml at 6 months postoperatively, and further to 8.15 ± 0.99 ml 1 year later, reflecting a significant and ongoing decrease (*p* < 0.001) [17]. Supporting these findings, Singh et al. found ultrasonographic signs of PCO in 77% (14/18) of women before bariatric surgery, with complete resolution in 55% (4/7) of these women at the one-year follow-up.However, in a study by Christ and Falcone, which evaluated 44 women with PCOS and 65 controls, ovarian volume did not show a significant decrease after surgery in either group [15]. This suggests that the effects of bariatric surgery on PCOS may vary depending on patient population and study methodology. Research suggests that bariatric surgery may lead to reduced insulin resistance, improved hirsutism scores, and enhanced menstruation and ovulation in women with PCOS [18]. In addition to these beneficial effects, alterations in reproductive hormones, particularly anti-Müllerian hormone (AMH), are also of great importance. In our study, no statistically significant difference was observed between preoperative AMH levels and those measured 3 months after bariatric surgery, regardless of PCO appearance on ultrasound. However, at 6 months post-surgery, AMH levels showed a significant increase compared to both the preoperative period and 3rd month postoperative levels. A similar study by Pilone et al. analyzed AMH values in 53 patients before sleeve gastrectomy and at 3 and 6 months postoperatively, finding no significant change between baseline and three-month values, but a notable rise in AMH levels 6 months after the operation [19]. In contrast, a study by Lv et al. reported a decrease in AMH levelsfollowing bariatric surgery [15]. Additionally, Merhi et al. examined AMH levels in 16 women before and after bariatric surgery, noting a significant reduction in patients under 35 years of age, while no decrease was observed in women older than 35 years [20]. The disparity in findings across these studies may be attributed to factors such as age, the presence of PCOS, and individual physiological responses to surgery. Therefore, while AMH levels provide valuable insights into ovarian reserve and function, they should not be interpreted in isolation.

The reduction of insulin resistance due to bariatric surgery may influence the adrenal gland’s effect on steroid hormone production. In the study by Sarwer et al., DHEA-S levels significantly decreased by the end of the second year after bariatric surgery, but no significant difference was noted at the end of the first year or between the first and second years [21]. In our study, when comparing DHEA-S with the preoperative value, we observed a significant increase in the 3rd and 6th postoperative months. This difference indicates that the response of DHEA-S levels to bariatric surgery may evolve over time, with potential increases in the short-term followed by decreases in the long-term. In the study by Ernst et al., a decline in DHEA-S hormone levels was noted in women who experienced significant weight losses following bariatric surgery [22]. Conversely, Ram et al. reported no change in DHEA-S plasma levels despite weight loss after laparoscopic adjustable gastric banding (LAGB) surgery [23]. The increase in DHEA-S seen in our study underscores the complexity of bariatric surgery’s effects on the hormonal system. This scenario highlights the need for long-term follow-up studies to gain a deeper understanding of hormonal changes after bariatric surgery over time. Variations in patient populations across studies (e.g., age, ethnicity, baseline DHEA-S levels, prevalence of PCOS, etc.) may contribute to differences in hormonal responses.

Bhandari et al. found that bariatric surgery was highly effective in eliminating hirsutism in obese women with PCOS [21]. In the majority of patients (74.6%), hirsutism was completely resolved within 6 months, with significant improvements observed over the following years (follow-up period: five years) [24]. Similarly, Singh et al. investigated the effects of bariatric surgery on clinical, biochemical, and hormonal parameters in women with PCOS and reported complete resolution of hirsutism in 44% of patients (5 out of 11), with the mean hirsutism score decreasing from 11 to 9 after 1 year [16]. In our study, the 6th postoperative month observed a 43% reduction in hirsutism. Extending the follow-up period and quantifying the degree of improvement in hirsutism could further enhance the comprehensiveness of our study and provide a more robust comparison.

Obesity is also associated with higher rates of dysmenorrhea, premenstrual disorders, and heavy menstrual bleeding. In a study by Pilone et al., AMH levels were found to increase following laparoscopic sleeve gastrectomy in obese women, and this increase was accompanied by improvements in the menstrual cycle and a reduction in dysmenorrhea [19]. Similarly, our study observed a statistically significant decrease in dysmenorrhea at 6 months. Obesity may contribute to chronic inflammation, which can elevate the production of molecules such as prostaglandins that play a role in dysmenorrhea. Weight loss may help lower prostaglandin levels by reducing inflammation, potentially leading to decreased menstrual pain.

This prospective observational study demonstrated that bariatric surgery leads to significant reductions in weight and body mass index in obese women, along with concurrent decreases in androgen levels (androstenedione and total testosterone) and increases in SHBG, DHEA-S, and AMH levels. Additionally, while this study did not directly assess fertility outcomes such as conception rates or time to pregnancy, the observed improvements in key reproductive parameters—specifically the reduction in hyperandrogenism (evidenced by decreased testosterone and androstenedione, normalization of ovarian morphology (reduced PCO appearance)—are strongly indicative of an enhanced endocrine environment conducive to improved ovulatory function and, consequently, fertility potential. Indeed, the restoration of regular ovulatory cycles and improved hormonal balance following bariatric surgery-induced weight loss are well-documented mechanisms leading to increased rates of spontaneous conception and reduced infertility in women with obesity, particularly those with PCOS [4, 5, 25]. The increase in AMH observed in our study at 6 months, alongside these other positive changes, may also reflect an improvement in the follicular environment, although its direct implication for fertility outcomes requires further nuanced investigation [26]. A limitation of this study is the lack of free testosterone measurements. While we observed significant changes in total testosterone and SHBG, the calculation of free testosterone, which requires serum albumin levels (not systematically collected for this purpose in our study), would have provided a more direct measure of bioavailable androgen. Future research should aim to include this parameter for a more comprehensive assessment of androgen status.

A further limitation is the reliance on BMI as the primary measure of obesity and weight loss, without detailed body composition analysis (e.g., fat mass, visceral adiposity). While BMI is a standard clinical tool, direct measures of fat mass and its distribution could offer more precise insights into the relationship between adipose tissue reduction and the observed hormonal and ovarian changes, particularly given the strong link between central adiposity and hyperandrogenism [27].

These findings highlight that bariatric surgery not only facilitates weight loss but also contributes to hormonal, clinical, and radiological alterations. In conclusion, bariatric surgery is an effective treatment option that may positively influence reproductive health by enhancing hormonal balance in obese women. However, further comprehensive studies are needed to evaluate its long-term effects and implications across different patient populations.

## Data Availability

No datasets were generated or analysed during the current study.
